# Sampling Methods for Metocean Data Aiming at Hydrodynamic Modeling of Estuarine and Coastal Areas

**DOI:** 10.3390/s20061732

**Published:** 2020-03-20

**Authors:** Jose Otavio Goulart Pecly, Paulo Cesar Colonna Rosman, Carlos Eduardo Parente Ribeiro

**Affiliations:** 1 AECO-COPPE/UFRJ, Centro de Tecnologia, Bloco C sala 209, Ocean Engineering Program-COPPE, Federal University of Rio de Janeiro, Rio de Janeiro RJ 21941-901, Brazil; pccrosman@ufrj.br; 2 AECO-COPPE/UFRJ, Centro de Tecnologia, Bloco I sala 104E, Ocean Engineering Program-COPPE, Federal University of Rio de Janeiro, Rio de Janeiro RJ 21941-901, Brazil; parente@peno.coppe.ufrj.br

**Keywords:** sampling, data acquisition, mathematical models, estuaries and coasts

## Abstract

Field observations require adequate metocean data gathering to promote the link between environmental diagnostic and prognostic obtained from modeling techniques. In general, model confidence can be improved by using data which present better quality and by improved parametrizations. This paper discusses and suggests timing routines for data gathering which are enough to describe the hydrodynamic behavior of estuarine and coastal areas. From the environmental diagnostics viewpoint, a sampling procedure is defined to the temporal scales providing data with adequate resolution to describe the natural process without signal aliasing. The proposed sampling procedure was based on the analysis of a data set of tides, currents, waves, water temperature, and meteorological variables observed at several stations along the Brazilian coast. The instrument setup was based mainly on the results of the harmonic analysis of tides. It is shown that the setup of instruments for simultaneous measurements of currents and waves requires special attention particularly in sites that present low currents and the action of waves. A subset of data gathered in shallow bays was used to estimate the surface turbulent stress by using a classical and a slightly modified parametrization for the wind drag coefficient. Under near neutral atmospheric stability conditions and high tide excursion, the surface turbulent stress obtained with the classical and the modified parametrization differed but the current profiles are expected to be only partially affected by wind-induced drift currents.

## 1. Introduction

Advances in electronic devices and manufacturing processes allowed developing sensors and sensor systems that are compact, accurate, and present low energy consumption. Powerful software interfaces and communication subsystems help the planning of standalone or telemetry-based stations and contribute to the widespread monitoring of natural water bodies.

Field observations ranging from relatively simple and over short periods [1] to long-term monitoring with large instrument networks [2] have been used to describe the movement of water bodies as well as for modeling studies [3,4].

The uncertainty of modeling studies can be attributed to input variables and parameters and can arises from modeling errors as well as from the sampling and measurement processes [5].

Although synoptic metocean monitoring is broadly reported in the literature, an extensive discussion on the spatial and temporal scales and their relationships with the instrument setup and timing is not usually found. The terminology metocean data refers to meteorological and oceanographic conditions.

As a result of inadequate timing, errors due to aliasing or due to non-stationary signals can affect the uncertainty of models that use such signals as contour conditions or as reference for the model calibration process.

The link between monitoring and modeling tasks can be established through the mathematical description of the movement of natural water bodies by the Reynolds Averaged Navier–Stokes equation (RANS). A brief analysis of the RANS equation and the turbulent stress parameterization indicates a minimum set of metocean variables that should be monitored in order to fulfill the validation and calibration requirements of hydrodynamic models.

Hydrodynamic models are considered as a fundamental tool to support research and engineering design of coastal facilities and structures. The model performance is evaluated comparing the agreement between model results and observed data. Even for models with good reported performance, the ability to reproduce the movement of water bodies depends heavily on the quality of the data gathered in the study area. Low quality data induces uncertain model results which may lead to non-correct design parameters.

As a continued goal, researchers and practitioners should follow procedures to gather environmental data that are based on the adequate spatial and temporal sampling scales. Efficient monitoring is endorsed by most monitoring initiatives quoting the “Ocean Best Practices” supported by The Global Ocean Observing System (GOOS). From a data gathering viewpoint, the goal of this study is to define a sampling procedure based on the scales associated with hydrodynamic processes in shallow waters. The description of such natural systems is usually obtained through large scale modeling techniques and turbulence parameterizations based on the Reynolds decomposition.

### Time Scales and Convergence

By the Reynolds decomposition, turbulent flux is described by a large scale and a small scale component representing the average value of a variable and its fluctuations, respectively. The decomposition of a variable *u* is then written as:

(1) $$u_{\;i}(t)\;=\;\bar{u_{i}(t)}\;+\;u_{i}{}^{\prime }(t)\;[i=1,\;2,\;3],$$

where $\bar{u_{i}(t)}$ denotes the time average of *u* and $u_{i}{}^{\prime }(t)$ represents its fluctuations.

The averaged value of the fluctuations $\bar{u_{i}´}$ is equal to zero for an infinity integration period *T* which, naturally, is not feasible. Then, the accuracy level will be determined by the integration period *T* which implies in an error due to finite averaging time.

The integral time scale ℑ is defined as the time that elapses until a signal presents no more correlation with itself and can be evaluated from the normalized autocorrelation function ρ(τ) by the expression $ℑ\;\equiv \;\int_{0}^{\infty }\rho \;(\tau )\;d\tau$. 

When the integration period *T* is much longer than the integral time scale ℑ, the root mean square error $\bar{\xi^{2}}$ between the real mean value and the observed mean value for $u_{i}(t)$ can be estimated by [6,7]:

(2) $$\bar{\xi^{2}}\;\approx \;2\;\frac{ℑ}{T}\;\bar{\;u_{i}\;{\left(t\right)}^{2}},$$

where $\bar{u_{i}\;{\left(t\right)}^{2}}$ represents the process variance.

The underlying assumption is that the process stationarity is kept during the integration time *T* and attention should be paid to this fact during the instrument setup for data gathering.

The Reynolds decomposition as depicted in Equation (1) allows a simplification of the Navier–Stokes momentum equations which are basic for this discussion.

## 2. Variables of Equations for Shallow Water Modeling and Instrumental Support

For the hydrodynamic modeling of an incompressible fluid and considering large scales, the Reynolds Averaged Navier–Stokes momentum equation for shallow waters can be simplified expanding the pressure term with the Boussinesq approximation and considering that the dynamic pressure and the spatial variations of the atmospheric pressure are very small. The momentum equation can be written, eliminating lower order terms, in order to obtain the following form [8]:

(3) $$\frac{\partial u_{i}}{\partial t}\;+\;u_{j}\frac{\partial u_{i}}{\partial x_{j}}\;+\;w\frac{\partial u_{i}}{\partial z}\approx -\;g\frac{\partial \eta }{\partial x_{i}}-g\int_{z}^{\eta }\;\frac{\partial (\rho /\rho_{0})}{\partial x_{i}}dz\;+\;\frac{\partial \;}{\partial z}\left[\frac{\tau_{i3}^{T}}{\rho_{0}}\right]\;+\;a_{i}$$

where the variables, suppressing overbars for simplicity, are average values and, using indicial notation, (*x*, *y*, *z*) ≡ (*x*_1_, *x*_2_, *x*_3_) and (*u*, *v*, *w*) ≡ (*u*_1_, *u*_2_, *u*_3_); in this way *u*, *v* and *w* are components of a velocity vector in the directions *x*, *y* e *z*; the variable *η* is the free surface level, ρ is the fluid density and ρ_0_ is a reference value for the density, the term $\tau_{i3}^{T}$ represents the turbulent stress; and the acceleration terms *a_i_* represent Coriolis forces.

The left side of Equation (3) represents the acceleration and advective terms. Vertical current profiles can be measured, in a selected point of the modeled domain, by using Acoustic Doppler Current Profilers (ADCPs). The term *w* ∂*u*/∂*z,* representing the gradient of the vertical velocity, is usually not accounted for in modeling due to its typically low values, and due to measurement noise and bias superposed. Researchers should pay special attention to the ADCP setup for gathering good and representative data of the water current time scales.

On shallow waters, the variations of the free surface level *η* play a very important role in water movement, which implies gathering tidal data by quality tidemeters. This fact is particularly important as surface water level variations are used as contour conditions at open boundaries. In such model limits, the readings of instruments based on pressure sensors installed at the bottom for measuring water level are influenced by the atmospheric pressure. This fact requires the simultaneous monitoring of atmospheric pressure variations in nearby located meteorological stations.

For rivers and channels affluent to the modeled domain, one should prescribe flow velocity or discharge rate [8]. For stream gauging, most common instruments for water velocity measurement employ mechanical, acoustic, and stage-based volumetric techniques [9] but also include systems based on imaging techniques. The transmission of water level sensors data based on IoT technology help to establish operational monitoring networks [10], which can also serve to modeling purposes.

The observed gradient of horizontal and vertical profiles of the mass density *ρ* = *ρ* (*S*, *T*, *p*), that is a function of the salinity *S*, temperature *T* and pressure *p*, defines the need of barotropic or baroclinic models. For coastal areas with small salinity variations, the water column stratification depends mainly on the water temperature and the current magnitude. We quote the instruments based on thermistors strings as a useful tool to gather time series of temperature profiles.

Considering the importance of the Coriolis forces, for small size domain models, the terms *a_i_* can be neglected.

Specific contour conditions result in turbulent stress represented by $\tau_{i3}^{T}$ which is related to variables that can be solved and, as so, is described by parameterizations at the bottom $\tau_{i}^{B}$ and at the surface $\tau_{i}^{S}$.

### 2.1. Parameterization of the Turbulent Stress Term

The turbulent stress at the bottom $\tau_{i}^{B}$ is commonly parameterized by the formulation of [11], when 2D and 3D models are coupled:

(4) $$\tau_{i}^{B}=\;f\;(\rho ,u_{*\;},H,\varepsilon ,U_{i})\;\left[i\;=\;1,\;2\right]$$

where *ρ* is the mass density, in kg m^−3^; *g* the gravity acceleration, in m s^−2^; u_*_ is the stress velocity, acting as a scale factor, in m s^−1^; *H* is the local depth, m; *ε* is the bottom roughness, m; and *U_i_* is the vertically averaged water speed in the *x_i_* direction, m s^−1^.

By using an Acoustic Doppler Current Profiler (ADCP), researchers can acquire data that describe current magnitude and direction along the water column; current profile data are processed to calculate the vertically averaged water speed *U_i_* which is used for evaluating Equation (4); the ADCP data is also used for calibrating the model, based on the comparison between modeled and observed values; considerations about the setup of ADCPs are found in Section 3 and in Section 5.3.

The turbulent stress on the surface $\tau_{i}^{S}$ is parameterized as a function of the wind speed described by:

(5) $$\tau_{i}^{S}=\;\rho_{air}\;C_{D}\;{\left(W_{10}\;-\;W_{0}\right)}^{2}\;\cos\;\varphi_{i}\;[i\;=\;1,\;2]\;,$$

where *ρ_air_* is the air density, in kg m^−3^; *C_D_* is the wind drag coefficient; *W_10_* is the wind speed 10 meters above surface, m s^−1^; *W_0_* is the wind speed at sea surface, m s^−1^; and *φ_i_* is the angle between the wind vector and the *x_i_* direction.

This formulation for $\tau_{i}^{S}$ uses a surface speed *W_0_* dependence and it has been shown to lead to improvements in models of tropical ocean waters [12,13].

The dependence of wind stress on the drag coefficient *C_D_* near the coastal zone was studied by [14] considering the neutral drag coefficient *C_DN_* over a variety of conditions. For non-neutral conditions, an atmospheric stability function *ψ_u_* (*ζ*) should be included to obtain the drag coefficient *C_D_* under observed conditions using [15]:

(6) $$C_{D}=\;{\left(C_{DN}^{-\;1/2}\;-\;\frac{\psi_{u}\;(\zeta )}{k}\;+\;\frac{1}{k}\;\ln\frac{C_{DN}}{C_{D}}\right)}^{-\;2},$$

where *ζ* is the stability parameter; and *k* is the von Kármán constant.

For modeling coastal areas, it is common practice to use a parameterization of the wind drag for neutral atmospheric stratification, which implies *C_D_* = *C_DN_*, expressed by [16]:

(7) $$C_{DN}=\;[0.80\;+\;0.065\;(W_{10}-W_{0})]\;\times \;10^{-3}$$

A minor modification of the above expression was made in the usual form by including the value of the wind speed *W_0_* at sea surface. Despite its intuitive importance, this term refers to a parameter that cannot be observed in situ.

### 2.2. Turbulent Stress and Free Surface Water Variation

Dealing with the atmospheric stability parameter [17], the solution for turbulent stress on the surface $\tau_{i}^{S}$ depends on solving an equation system that describes aspects of air–sea interaction [18].

For evaluating this interaction, the COARE algorithm [19] solves the atmospheric stability functions. Please, note that the COARE algorithm documentation and the newest Fortran and Matlab computer codes can be accessed at Flux Documentation-Ocean Climate Stations [20]. The bulk COARE algorithm requires input data of wind speed, air temperature and relative humidity, solar irradiance, and precipitation; sea surface temperature and water temperature 6 m below the surface are also needed. These variables are not usually observed and represent a limitation to algorithm application. This aspect implies the calculation of the turbulent stress with the help of parametrizations like the expressed in Equation (7) which depends only on the wind data corrected to a reference height. As a usual recommendation, the standard is to gather wind data at 10 m above the surface, and the temperature and relative humidity are measured 2 m above the surface. As the water level *η* changes with time, such reference heights are not maintained, and some form of height transformation is needed.

For shallow water sites with significant tidal excursions, and when winds are not observed on the reference level of 10 m above the surface, the relative importance of the wind sensor mounting height should be evaluated, and corrections applied to turbulent stress terms. This evaluation, however, requires that a set of variables be available as described in the following section.

## 3. The Selected Set of Variables and the Need of Specific Sensors

Based on the discussion presented so far for shallow waters, it is possible to select a representative set of metocean variables with two purposes: (i) to allow a hydrodynamic circulation analysis indicating the relationship between cause and effects, and (ii) to provide elements for model validation or calibration.

**Figure 1:**
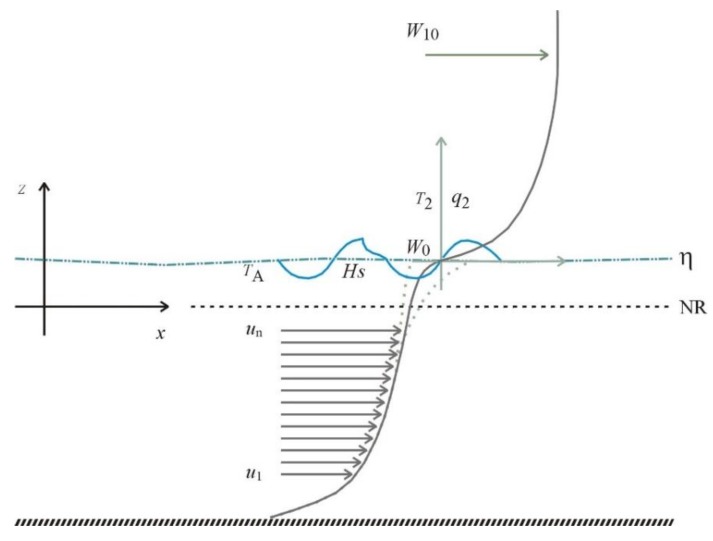
Combined profiles of currents and winds obtained from equations iteratively solved with the measured data.

As indicated by the previous discussion, specific sensors and sensor systems should be used to gather data of: water level *η* referenced to a vertical datum NR, water current profile *u_n_*, wind *W_10_* measured 10 m above surface, wind *W_0_* measured at the surface, water temperature profiles, water temperature *T_A_* at the surface, temperature *T_2_* and air specific humidity *q_2_* measured 2 m above the surface, atmospheric pressure *p_a_* and, whenever possible, the main wave parameters: significant height *H_S_*, peak period *T_P_* and associated direction *D_P_*. Some of these variables are depicted in Figure 1 which also illustrates the matching between the water current profile and the wind profile.

At the air–sea interface, part of the wind energy is transferred to the water surface and induces the wind–wave orbital velocities while other part induces drift currents. For the purposes of this paper, we are dealing with time-averaged values. The expected residual values of the orbital velocities should be small in comparison with the vertically averaged current magnitude. On the other hand, under low to moderate-to-strong wind speed, drift currents are small in comparison with the vertically averaged current magnitudes particularly under moderate-to-strong currents. The typical ratio between the surface drift and the wind speed is reported as about 3% [21].

Aiming at a simplified solution, a continuity condition at the free surface between the wind and current profiles is imposed by the method proposed in this paper. This condition is represented by *W_0_* that, due to practical limitations, cannot be measured in situ.

An iterative procedure should be implemented in such a way that the current *u*_0_ at the surface approximates, to a certain threshold, the value of wind speed *W_0_* evaluated at the surface.

For neutral atmospheric conditions, the main steps of the procedure are:

1. Let the first non-contaminated ADCP layer (please refers to the label *u_n_* in Figure 1) be an estimation for *W_0_* and correct the wind speed *W_Z_* observed at height *z* by using:
  (8) $$\frac{W_{10}-W_{0}}{W_{Z}-W_{0}}\;\approx \;{\left(\frac{10}{z-\eta }\right)}^{\frac{1}{7}},$$
2. Calculate the turbulent stress on the surface $\tau_{i}^{S}$ and at the bottom $\tau_{i}^{B}$;
3. Solve the water current profile (analytical or numerical) and let the water current at the surface *u*_0_ be a new estimation for *W_0_*;
4. Repeat steps (2) and (3) until convergence.

As previously explained, the observed variables included in the momentum equation are large scale variables and, under the hypothesis of an ergodic process, the time averaged values are a good approximation for the ensemble average [7].

## 4. Ensemble Averaging

**Figure 2:**
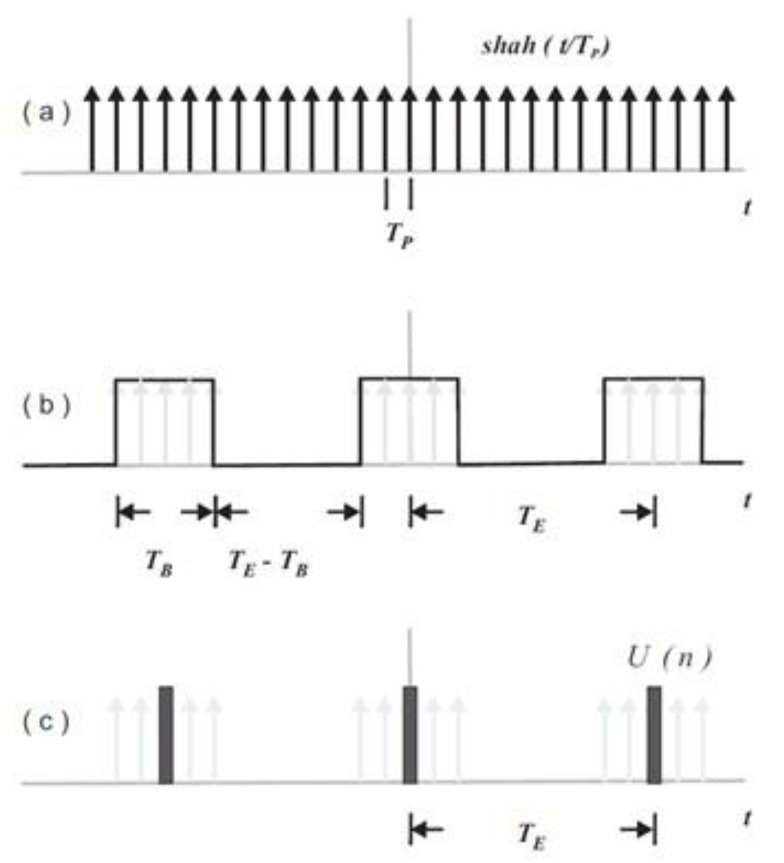
Ensemble averaging obtained by: (**a**) uniform sampling; (**b**) time windowing; and (**c**) ensemble value calculated during period *T_B_* resulting in low-pass filtering.

The diagram presented in Figure 2 is used to understand the effect of ensemble averaging on a continuous variable on time *u*(*t*). Figure 2a shows a unit impulse train (function *shah*) used to take samples of *u*(*t*) at uniform time intervals *T_P_* by a multiplication process. The discrete-time sequence *u*(*k*) obtained with the sampling process is multiplied by a periodic pulse train with a width of *T_B_* and a period *T_E_* as shown in Figure 2b. Within the time *T_B_* a set of 2*N* + 1 samples is averaged according to weighting factors and the resulting value is registered at time intervals *T_E_* as an estimation of the average value of the signal *u*(*t*) along the ensemble time as shown in Figure 2c.

The estimation *U*(*n*) for the analog function average value $\bar{u_{i}}$ can then be represented by the symmetric low pass digital filter with 2*N* + 1 weighting factors as [22]:

(9) $$U[n]\;=\;\frac{1}{2N+1}\;\sum_{k=-N}^{N}\;u\;[n+k],$$

Naturally, the number of samples 2*N* + 1 and the associated burst time *T_B_* will determine the degree of smoothness of the ensemble sequence and will also determine an amplitude error for time-varying signals. In other words, there is a tradeoff as a quite long burst time *T_B_* will result in loss of process stationarity.

A study on ensemble timing is presented in the following section to cover the requirements for turbulence filtering and for avoiding aliasing errors.

## 5. Sampling Procedure for Metocean Variables

Ensemble timing defines the time interval for the time between pulses *T_P_*, for the burst duration *T_B_* and for the ensemble time *T_E_* with the following restrictions and requirements: (i) the ensemble time *T_E_* is determined by the Nyquist–Shannon to avoid aliasing on flow patterns modulated by tides; (ii) the burst time *T_B_* is adjusted to the need of keeping the process stationary during the ensemble sampling; (iii) the time between pulses *T_P_* is determined by the intended smoothness degree; the high frequency content of wind waves has to be accounted when measuring waves parameters.

Aiming to determine a rule for the ensemble timing and the record length or the monitoring period, a dataset from monitoring stations along the Brazilian coast was analyzed to identify the characteristic time scales. The main results are shown below.

### 5.1. Timing for Tide Monitoring

For a time varying sinusoidal signal representing a tide constituent with amplitude A and frequency *ω* = 2π*f*, the maximum slew rate *ΔN*/*ΔT* occurs at zero crossing and is expressed by:

(10) $$\frac{\Delta N}{\Delta T}=\frac{d}{dt}\;A\;{sin\omega t|}_{t=0}=A\omega$$

The resulting error *ε*, written as a fraction of the peak-to-peak variation due a finite acquisition time *ΔT* = *T_B_*, is estimated by the slew rate at zero crossing given by:

(11) $$\varepsilon =\;\pi \;f\;T_{B}$$

For the free surface water level *η,* the analyses presented in this paper were carried out in tidal time series freely available [23] from the University of Hawaii Sea Level Center [24]. This online repository includes information about the GLOSS (The Global Sea Level Observing System) station number, geographical coordinates, and hourly and daily records with verified quality. A one-year dataset from 8 stations from south to north of the Brazilian coast was selected to described different tidal patterns due to its geographic location distant from each other. The selected stations are: Rio Grande (GLOSS Number 193), Imbituba (GLOSS Number 351), Ilha Fiscal (GLOSS Number 195), Salvador (GLOSS Number 334), Fernando de Noronha (GLOSS Number 198), Fortaleza (GLOSS Number 336), Madeira (GLOSS Number 200), Belem (JASL Number 229a).The dataset from the 8 selected stations was analyzed through Fast Fourier Transform (FFT) algorithms and through harmonic analysis and prediction routines found in the IOS Tidal Package [25], whose documentation and Fortran code is available [26].

On the selected stations, the harmonic constituent with the highest frequency and significant amplitude was M8, which has a period of about 3.1 h. Applying Equation (12), the burst period *T_B_* should not be longer than 3 min to keep the full-scale error *ε* within 5% for the M8 constituent. Although a percentual error of 5% seems to be high, it was defined considering that the M8 constituent presented an amplitude of less than 2 cm for sites in southern Brazilian coast. For a station inside Todos os Santos Bay, at mid-latitudes, the M8 constituent reached an amplitude of 6.5 cm; overtides with higher amplitude may be observed in specific sites. For those stations on the northern Brazilian coast, the M6 and M4 constituents presented amplitude of about 5.5 and 13.5 cm, respectively, leading to burst periods of 4 and 6 min under the same considerations.

Setting the sampling period to four times the Nyquist–Shannon period, the sampling period *T_E_* should be shorter than 25 min for the M8 constituent and 30 min for the M6 constituent.

In order to evaluate the quality of contour conditions for modeling based on short tide records, one-month registers around the equinoctial tide were also analyzed for the same stations. For stations with small meteorological effects, the mean sea level estimated from the annual series differed within ± 2 cm from the monthly series. The lowest astronomical tide (LAT), however, differed up to 15 cm when estimated from the annual or the monthly register.

Additionally, gaps were introduced within the monthly series to evaluate the possibility of using still shorter registers to represent the water level as a contour condition. With the benefits of gap handling capability of the Foreman´s routines [25], it was possible to replace the registers of a period no longer than the final 6 days of a monthly time series with gaps, while keeping nearly the same mean level and providing a similar set of harmonic constants.

### 5.2. Water Temperature—Time Scales in a Shallow Bay

The water temperature dataset for the Guanabara Bay (located in Rio de Janeiro) was selected because it is the only average-to-long-term dataset that could be identified along the Brazilian coast. Additionally, this dataset can be correlated with the tidal data from the Ilha Fiscal station, one of the selected stations for tidal analysis, located inside the Guanabara Bay.

**Figure 3:**
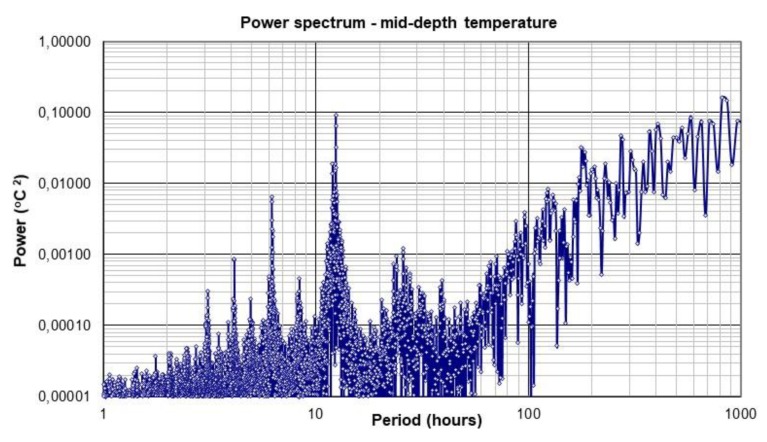
Power spectrum for mid-depth water temperature acquired inside the Guanabara bay; the local depth is 21 m.

Water column temperature profiles were studied with the data gathered at 30 min intervals by Aanderaa TR-7 thermistor strings installed inside the Guanabara Bay, coordinates 22.87 S and 43.15 W during a period of about 8 months in a 21 m deep site [27]. Considering that the inner water temperatures are dependent on the solar radiation and air temperature, the water temperature monitored was strongly affected by the flushing of the bay due to tides; temperature profiles strongly influence the dilution pattern in these sites [28]. Figure 3 shows the power spectrum for the mid-depth water temperature gathered along 8 months and depicts energy peaks with periods of about 25.8 h (O1), 12.4 h (M2, with the highest energy), 8.3 h (M3), 6.2 h (M4), 4.1 h (M6), and 3.1 h (M8, with low energy) which are about the same of the above-cited tidal constituents.

Due to the strong tide modulation in the temperatures observed, it is proposed an ensemble timing similar to the proposed for tide monitoring.

The density stratification and the current profile define the degree of stratification for the water column. Based on this statement, it is a good practice to sample water temperature and currents at the same depth levels whenever possible.

### 5.3. Monitoring of Current Profiles and Waves

The most common form of using an Acoustic Doppler Current Profiler (ADCP) in coastal waters is the upward-looking fixed mooring which allows simultaneous measurement of the waves and current patterns. The ADCP used was a Workhorse Waves Array operating at 600 kHz manufactured by Teledyne RDI. The Waves Array is an ADCP for current profiling, which also gathers data about wave-induced orbital velocities by the addition of a pressure sensor and a specialized firmware. The instrument was deployed at the bottom attached to a double-axis gimbal to assure alignment very close to the vertical. Please note, the vertical alignment is a requirement for gathering good quality data, particularly for wave measurement.

**Figure 4:**
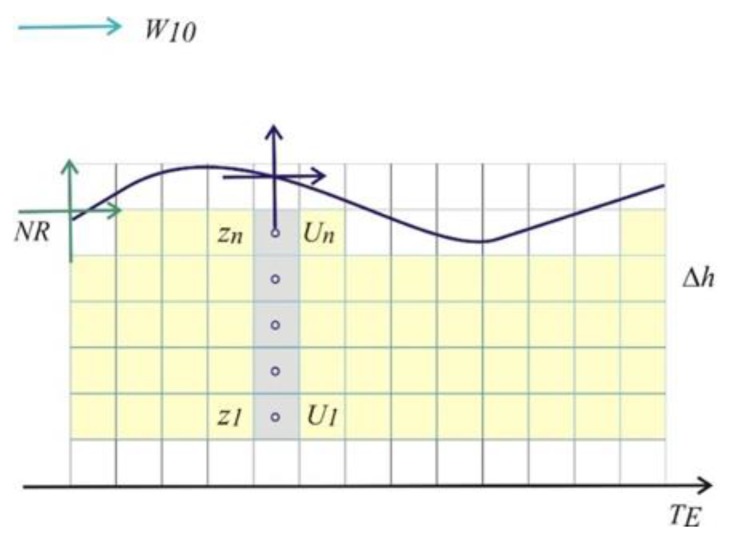
Current profiles for a range of depths from near the bottom to near the surface; the shallower ADCP layer and the wind sensor reference height depends on tidal variation.

The ADCP can do the measurement of the water current at discrete bins with size *Δh* according to a user-defined setup. Figure 4 shows the wind speed *W_10_,* the vertical reference *NR* of the model, the depth layer size *Δh*; the instrument yields a velocity profile for a range of depths from near the bottom (*U_1_* at *z_1_*) to near the surface (*Un* at *z_n_*). Current profile data are represented by averaging a set of profiles at ensemble interval *T_E_*.

The time between pulses *T_P_* is the repetition rate of acoustic pulses into the water and is related to the high-frequency content of the water level signal. It was found that values of *T_P_* higher than 2 s imply the need for a long averaging time for the ensemble due to the interaction with the waves field. Consequently, the resulting burst time *T_B_* will be longer, and the slew-rate error will be higher. Once defined the value for *T_B_*, the number of pulses 2*N* + 1 will define the burst time and will be inversely related to the standard deviation of the averaged ensemble value. The depth cell size *Δh* is also inversely related to the standard deviation of the measurement.

#### Effect of Waves on Current Ensembles

The influence of the sea state on the average value of the current ensembles was evaluated with the data of an ADCP Waves Array 600 kHz deployed in two coastal stations with different wave climates. The instrument was deployed 4 km away from Rio de Janeiro State coast at a place 13 m deep and 2 km away from Bahia State coast at a place 26 m deep.

A typical setup for wave data acquisition uses a value of *T_P_* of half second and a burst time *T_B_* of 20 min. This value of *T_P_* seems adequate if we consider that wind waves present a period higher than 3–4 s. During a burst time of 20 min, the instrument will gather 2400 samples that are enough for high-resolution spectral analysis.

The time series of the horizontal component of the velocity sampled at 2 Hz were analyzed to evaluate the time scale associated with the convergence of its averaged values.

Equation (2) was evaluated for a relative error level of 2%, considering three sea states represented by increasing significant wave height *Hs*. The results are summarized in Table 1 for conditions of low, medium, and high values of vertically averaged current magnitude *U* observed during some selected events. The integration period *T* needed to obtain the defined relative error was calculated for three depth cells named *Bin 1*, *Bin 2*, and *Bin 3*, which form the Waves Array with *Bin* 3 closest to the sea surface.

As we discard very shallow ADCP depth cells due to secondary lobe acoustic contamination, the values associated with *Bin* 1 and *Bin* 2 were used as an estimation of the averaging time to be used for the current ensemble.

The results presented in Table 1 show that conditions of calm sea and high currents require the use of lower integration times. On the other side, severe sea conditions over low currents require higher integration times.

Then, the variance associated with the averaged value of the ensemble increases with the variance of the orbital velocity and with the decrease of the current magnitude.

**Figure 5:**
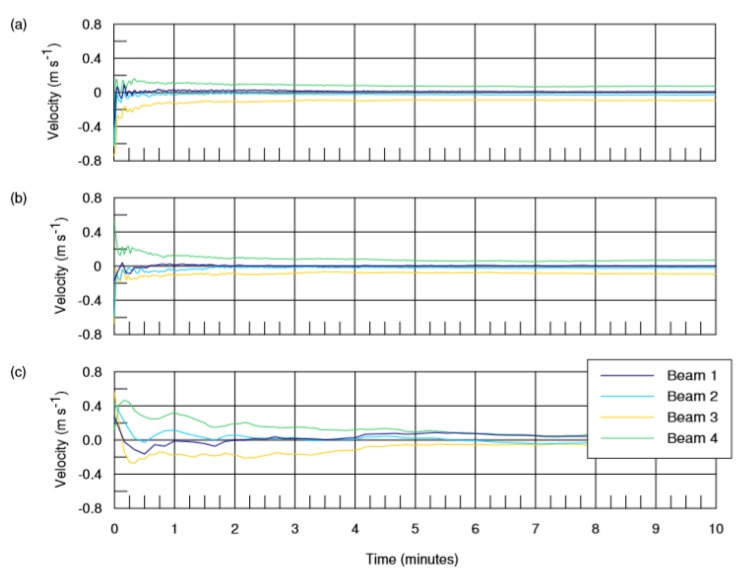
Time averaged values for the radial velocities time series resampled on lower rates of: (**a**) 1 Hz; (**b**) 0.5 Hz; and (**c**) 0.2 Hz.

In order to evaluate the influence of the interval between pulses *T_P_* on the convergence of the ensemble average, the data of radial velocity time series for the events observed in Rio de Janeiro on Day 3 at 01:00 AM were resampled on rates of 1 Hz, 0.5 Hz and 0.2 Hz for the ADCP beams 1 to 4; the convergence of the time-averaged values is shown in Figure 5.

These results indicate that values of pulse interval *T_P_* higher than 0.5 Hz or 2 s do not lead to the convergence of the average value of the current ensemble, to a 5% threshold, over a period shorter than 3 min as quoted in Section 5.1. An additional consideration against higher *T_P_* values is the aliasing induced in the short period variations of sea level surface.

Near the surface, the magnitude of the current is affected by the wave-current interaction process and is strongly dependent on the wind pattern. The coupled solution for the current and wind profiles near the surface requires data of a set of meteorological variables.

### 5.4. Monitoring Meteorological Parameters

In order to estimate turbulent stress on surface $\tau_{i}^{S}$ winds, air temperature, air relative humidity and atmospheric pressure, whose sensors are commonly present in coastal meteorological stations, should be monitored.

The winds should be sampled with a time between pulses shorter than 5 s (ideally 1 s). The choice of the burst time *T_B_* is defined by the position of the spectral gap in the wind power spectrum presented by Van der Hoven [29]. This gap is a low energy frequency range corresponding to periods between 10 min and 1 h. In this frequency range the average wind speed presents some degree of stationarity, which means averaged values observed in periods between 10 min and 1 h are relatively stable.

Similar gaps were also identified in the air temperature and air humidity power spectra [30]; this fact suggests the use of similar setups for all meteorological variables.

Recommendations for the measurement of meteorological variables, as presented by WMO [31], are useful for research institutions and instrument manufacturers. Important aspects of station sitting and exposure are discussed. The general discussion found in the guide [31] indicates that wind records should be averaged over 10 min interval, by taking samples at intervals of 1 s, for forecasting purposes. The WMO Guide [31] also recommends that the wind profile dependence on atmospheric stability should be accounted for and advises to take samples at intervals of 0.25 s in case we need to determine wind gusts.

Field data gathered at 2 stations were selected for evaluating wind stress over the water surface, as presented in Section 6. One station was installed inside Ribeira Bay (south of Rio de Janeiro, approximate location 23.09 S and 44.40 W), and the second one installed inside Marajó Bay (approximate location 0.56 S and 47.91 W). A mechanical sensor model 05305 manufactured by RMYoung gathered wind data from the Ribeira station and the wind data from Marajó station was gathered by an acoustic sensor WindSonic manufactured by Gill. Both sensors were connected to Campbell Scientific CR1000 dataloggers operating in a standalone mode. The dataloggers were programmed to acquire data every 5 s and to record averaged values at 10 min intervals.

### 5.5. Summary of the Proposed Timing

The synoptic monitoring of the variables of interest allows identifying the relationship between causes and effects.

Considering the tidal modulation on the hydrodynamic pattern of coastal areas, the timing for gathering water level data is mainly defined by the temporal scales of the higher frequency tidal constituent. Overall, the timing proposed for the other metocean variables is derived from the timing proposed for tide monitoring. As a rule, the timing for tidal currents should follow the setup for water level data gathering. In a typical coastal station for wind and atmospheric pressure measurement, the inclusion of additional sensors for air temperature and humidity, and solar radiation presents low overhead. It can be useful to study the atmospheric stability and to provide data for water quality models.

Table 2 presents a summary of the proposed setup for coastal and estuarine monitoring studies. Naturally, not all instruments allow full flexibility in setting the ensemble timing. On the other hand, the proposed minimum period of 24 days for data gathering aiming at modeling tasks relies on the capability of gap handling of the harmonic analysis tools, as previously discussed.

Please note that the selection of the preferred ensemble time *T_E_* as 10 min was also determined for coincidence with the timing for measuring winds (see Section 5.4). Naturally, an ensemble time *T_E_* of 20 min (see Section 5.3) allows data resampling aiming at cross correlation analysis between variables, in cases where low power consumption is priority.

### 5.6. Considerations about Power Consumption

The power consumption of meteorological sensors and controlling dataloggers and tidemeters is usually very low with current technology. The power consumption increases strongly for stations equipped with telemetry devices. In such cases, solar panels can usually provide energy for stations with no mains supply.

The concern is related to the power consumption of ADCP systems that need to operate in a standalone mode. For typical stations established with instruments for current measurement, the energy available from standard battery packs is usually enough for more than two months of continuous operation. The selection of an ensemble time *T_E_* of 20 min, as suggested in the previous section, will result in a deployment with the double of autonomy. For typical coastal stations, data quality should not suffer from this extended ensemble time. On the other hand, the use of higher sampling rates while keeping the same ensemble time *T_E_* increases power consumption and reduces the autonomy proportionally.

Based on the available technology, the quoted procedure does not pose restrictions regarding memory size or energy consumption except for the ADCPs monitoring wave data. The autonomy for current and wave measurements with a Workhorse Waves Array 600 kHz configured with the values proposed in Table 2 (*T_P_* of 1 s, *T_B_* of 3 min and *T_E_* of 10 min for currents; and *T_P_* of 0.5 s, *T_B_* of 20 min and *T_E_* of 1 h for waves) is about 30 days. Please note, these are typical values that can change depending on environmental factors, battery age, deployment depth, and different ADCP models. Naturally, the mooring autonomy concerning energy can be extended with the use of an external battery pack. In summary, we can carry out field works lasting up to 30 days, or even more, in order to gather good quality data.

## 6. Estimation of the Wind Stress on the Surface

Field monitoring was carried out with instruments programmed according to the timing setup described in this paper.

The typical setup was first considered for moorings on the shallow waters of Ribeira Bay located in the south of Rio de Janeiro State. The data indicated low wind speeds (average of 4.5 m s^−1^ not exceeding 9 m s^−1^ in short periods), small tidal excursions (lower than 1.5 m), low water currents (not exceeding 30 cm s^−1^) and different atmospheric conditions during the observation period. However, as no data was available for sea surface temperature and water temperature below surface, the bulk COARE algorithm was not applied.

Another evaluation was done with the data gathered near the mouth of Marajó Bay located on the northern Brazilian coast. The local depth was about 23 m. In this site, the maximum wind speeds were about 15 m s^−1^ during the observed period, tide excursion was about 5.0 meters in height and surface water currents reached 1.8 m s^−1^ after half-tide time.

**Figure 6:**
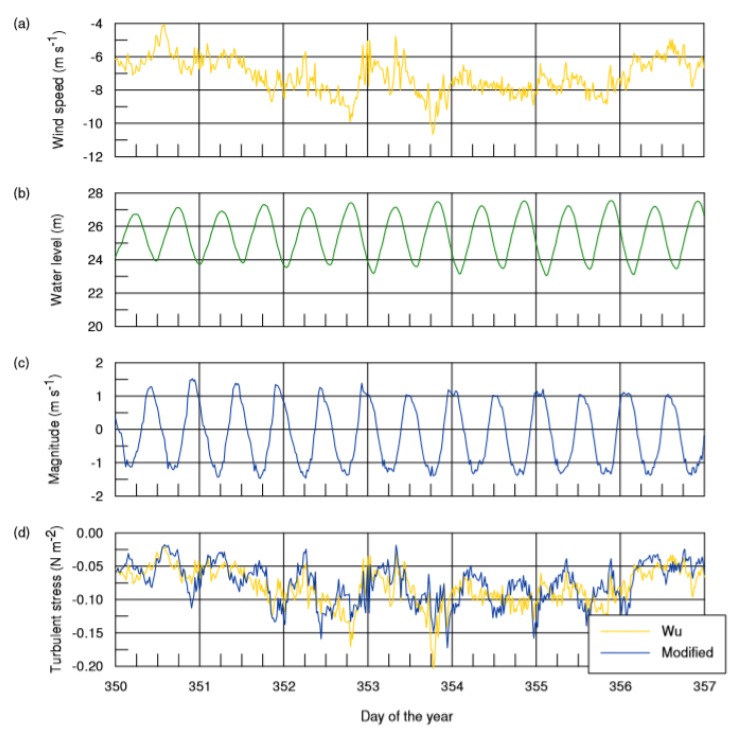
Marajó station time series for: (**a**) zonal wind speed, (**b**) water level variations, (**c**) zonal depth averaged current magnitude; and (**d**) a comparison between the zonal components of the surface turbulent stress obtained by using a neutral wind drag coefficient *C_DN_* and by using the modified method described in this paper.

When considering the near-neutral atmospheric conditions, instead of the application of an air–sea coupling model, turbulent stress values were calculated with the help of the Wu formulation [16]. The results were compared with the modified method by including the value of the wind speed *W_0_* at sea surface and *η* the free surface elevation. As exemplified in Figure 6, based on the Marajó station data for the zonal component of the turbulent stress, the values obtained with the two methods are different, as depicted in Figure 6d, but present the same tendency.

Under the action of strong winds and low tidal currents, the major differences between observed and depth-averaged current values are expected to occur at the bottom and the surface.

During the observed period, the winds of lower intensity occurred at the site with the lower tidal currents associated with the lower tidal excursion; on the other hand, the stronger winds occurred at the site with the higher tidal currents associated with the higher tidal excursion.

Although the modified parametrization generated different values of the wind turbulent stress over the surface, the high tidal currents observed at Marajó station are only partially affected by wind-induced drift currents.

For such site, which presents strong tidal currents, the description of currents by using 2D depth-averaged values should be appropriate, as a first guess, for estimating *W*_0_.

## 7. Conclusions and Recommendations

A set of metocean variables was defined as enough to solve a simplified form of the momentum equation and lead to a better understanding of the movement of the water on coastal and estuarine areas.

Generally, the hydrodynamic models require data of water level, water current profiles, winds, atmospheric pressure, and bed roughness data as a calibration parameter. Under near-neutral atmospheric stability, temperature and air humidity and water temperature data can be neglected.

Water speed on the surface *W*_0_ and free surface variation *η* were added in a wind drag formulation leading to a better representation of the natural process through the computational models. Additional research should be undertaken by running 3D computational models for large estuarine systems to compare the results obtained with the use of the classical [16] and with the modified formulation.

An ensemble setup whose timing is believed to be adequate for monitoring most of the sites located on shallow waters was proposed for a selected set of variables. The timing used is related to the free surface elevation signal as coastal and estuarine hydrodynamic circulation depends on tidal time scales. 

Overall, it is proposed to gather data on tides and tidal currents for 3 min over a 10-min interval. For sites that present low currents under severe sea conditions, the proposed timing is to gather data for 10 min over a 20-min interval, to decrease power consumption. For meteorological variables, we follow the standard recommendation for taking samples continuously over a 10-min interval, which is also coincident with the ensemble period for tides. 

As general guidelines for data gathering at coastal areas, we quote: (i) the approach based on the signal slew rate results in burst time *T_B_* shorter than 3 min for both water level and current signals; (ii) ensemble time *T_E_* for acquiring water temperature, water level, currents and winds and other meteorological variables should be defined as 10 min; (iii) for all variables, records should be acquired at 1 Hz rate, except for waves that should be acquired at 2 Hz; (vi) under low currents and severe sea conditions, the burst time *T_B_* should be increased to 10 min and the ensemble time T_E_ increased to 20 min; (v) the observation period aiming at modeling tasks should be longer than 24 days; (vi) the application of a bulk air–sea interaction algorithm is not practical for most engineering applications as it requires additional variables which are not usually available. With the current instrument technology, the typical setup should be adequate for field campaigns with duration of one month or even more; monitoring periods of at least one month encompass different tidal patterns and present a good probability for covering different meteorological conditions.

## Figures and Tables

**Table 1:** Values of the integration period for the ensemble of currents under different sea states. The stations comprise only one ADCP Workhorse 600 kHz for current and wave measurements.

Station	Time	*Hs* (m)	*U* (m/s)	Integration Period *T* (s)		
				*Bin* 1	*Bin* 2	*Bin* 3
Rio de Janeiro (location 21.82 S and 40.89 W at *h* = 13 m)	Bin depth (m) →			2.5	1.5	0.5
	Day 1, 02:00 PM	1.0	0.6	18	33	57
	Day 2, 04:00 AM	1.5	1.0	23	31	47
	Day 3, 01:00 AM	1.8	0.3	316	438	590
Bahia (location 13.05 S and 38.47 W at *h* = 26 m)	Bin depth (m) →			7	5	3
	Day 1, 09:00 PM	1.2	0.7	17	26	37
	Day 2, 02:00 PM	2.5	0.2	895	1330	1705
	Day 3, 07:00 PM	3.5	0.5	296	411	588

**Table 2:** Values of ensemble timing under different sea conditions.

Variable	Discretization	Condition/ Position	Timing			Monitoring Period
			*T_P_*	*T_B_*	*T_E_*	
Water level		Domain limits	1 s	3 min	10 min	24 days for modeling 1 year for diagnostic
Currents on moorings	Vertical profile with 10 or more levels		1 s	3 min	10 min	
		Low currents on severe sea		10 min	20 min	
Water temperature			1 s	3 min	10 min	
Winds		Height of 10 m above surface	1 s	10 min	10 min	24 days for modeling 1 year for diagnostic
Air temperature and humidity		Height of 2 m above surface				
Atmospheric pressure, solar radiation						
Waves		ADCP depth define low frequency cutoff	0.5 s	20 min	1 h	1 year for diagnostic and modeling

## Nomenclature

*u_i_*	water velocity
*η*	free surface level
ρ	fluid density
$\tau_{i}^{B}$	turbukent stress at the bottom
$\tau_{i}^{S}$	turbulent stress at the surface
*S*	salinity
*T*	water temperature
*p*	pressure
*H*	local depth
*U_i_*	vertically averaged current magnitude
*ρ_air_*	air density
*C_D_*	wind drag coefficient
*C_DN_*	neutral drag coefficient
*W_10_*	wind speed 10 m above surface
*W_0_*	wind speed at sea surface
*W_Z_*	wind speed at height z above surface
*ψ_u_* (*ζ*)	atmospheric stability function
*ζ*	stability parameter
*T_A_*	water temperature at the surface
*T_2_*	temperature measured 2 m above the surface
*q_2_*	air specific humidity measured 2 above the surface
*p_a_*	atmospheric pressure
*H_S_*	significant wave height
*T_P_*	wave peak period
*D_P_*	wave peak direction
*ω*	angular frequency
*T_P_*	sampling time
*T_B_*	burst time
*T_E_*	ensemble time
